# A Rare Case of Spontaneous Tumor Lysis Syndrome in Hodgkin Lymphoma

**DOI:** 10.7759/cureus.15887

**Published:** 2021-06-24

**Authors:** Moeez Hassan, Dilnaz Alam, Nadia Altaf, Asif K Kundi

**Affiliations:** 1 Pediatrics, Beaumont Hospital, Royal Oak, USA; 2 Internal Medicine, Saint Joseph Mercy Health System, Livonia, USA; 3 Pathology/Hematology, Khyber Medical University, Royal Oak, USA; 4 Internal Medicine, Leidos QTC Healthcare Services, Phildelphia, USA

**Keywords:** spontaneous tumor lysis without precipitating factor, tumor-lysis syndrome, acute spontaneous tumor lysis syndrome, hodgkin lymphona, cairo and bishop

## Abstract

Tumor lysis syndrome (TLS) is an oncological emergency characterized by biochemical abnormalities such as metabolic acidosis, hyperkalemia, hyperphosphatemia, and hypocalcemia. The clinical outcome is directly related to the biochemical abnormalities. TLS can occur in any malignancy, but it is highly associated with rapidly proliferating tumors. Although the syndrome is commonly associated with hematological malignancies, particularly with leukemia and non-Hodgkin’s lymphoma, it is rarely seen in patients with Hodgkin’s lymphoma. In our case, a 7-year-old girl presented with intermittent fever, non-productive cough, fatigue, and night sweats for four months. On examination, she had an enlarged cervical lymph node of 5 cm in size on the left side accompanied by palpable supraclavicular lymphadenopathy. Past medical history was significant for the relapsing and remitting course of nephrotic syndrome diagnosed two years before presentation. The patient underwent a left-sided cervical node excisional biopsy, which confirmed classical Hodgkin’s lymphoma of mixed cellularity type. Her baseline chest x-ray revealed a bulky anterior mediastinal mass. To stage the tumor, a bone marrow biopsy, CT, and positron emission tomography (PET) scan were done. Although the bone marrow biopsy report showed a normal pattern of trilineage hematopoiesis, the CT and PET scan results led to its classification under stage 4. During her stay in the hospital for further work-up and treatment, her condition suddenly deteriorated. There were biochemical derangements on lab reports that confirmed the Spontaneous Tumor Lysis Syndrome (STLS). She recovered completely due to immediate stabilization and correction of electrolyte abnormalities. STLS is a life-threatening condition that is rarely seen in patients with Hodgkin’s lymphoma. The treating physicians should be vigilant about this possible sequela of Hodgkin’s lymphoma and be aware of its different presentations.

## Introduction

Tumor lysis syndrome (TLS) is a common metabolic complication of certain hematologic malignancies. The rapid lysis of proliferating tumor cells releases intracellular substances in the extracellular fluid, which triggers the pathophysiology of TLS and its clinical outcomes. The onset of TLS is usually linked with the initiation of chemotherapy, but in some cases, spontaneous tumor lysis syndrome (STLS) could also ensue without prior chemotherapy exposure. TLS is commonly observed in high-grade hematological malignancies such as B-cell non-Hodgkin’s lymphoma after chemotherapy [1]. Previous literature reviews revealed that the possibility of STLS with hematological malignancies is rare (1.08%) [2]. The literature to support the mechanism of STLS is very limited. Previous studies have suggested that high levels of cytokines and hyperthermia cause tumor cell death. The risk is higher in patients with larger tumor sizes [3]. 

## Case presentation

A 7-year-old Asian female presented to our outpatient clinic with intermittent fevers and left-sided neck swelling. She had low-grade fevers (100.4^0^ F-102^0^F) recurring periodically over the last four months, accompanied by night sweats, fatigue, and weight loss. She had stopped attending school due to her illness. The painless swelling in her neck persisted for three months. However, few weeks prior to the presentation, there had been a gradual and progressive increase in its size. Her past medical history was significant for steroid-sensitive nephrotic syndrome diagnosed about two years ago, and since then, she has had frequent relapses of the disease. At the time of presentation, she was in remission for nephrotic syndrome and was not taking any medications. She also complained of abdominal pain, loss of appetite, dry cough, headache, and unintentional 3 kg weight loss in the past four months. She denied any history of chest pain, palpitations, dyspnea, visual changes, rashes, hematuria, urinary frequency, joint pain, and body aches.

**Figure 1:**
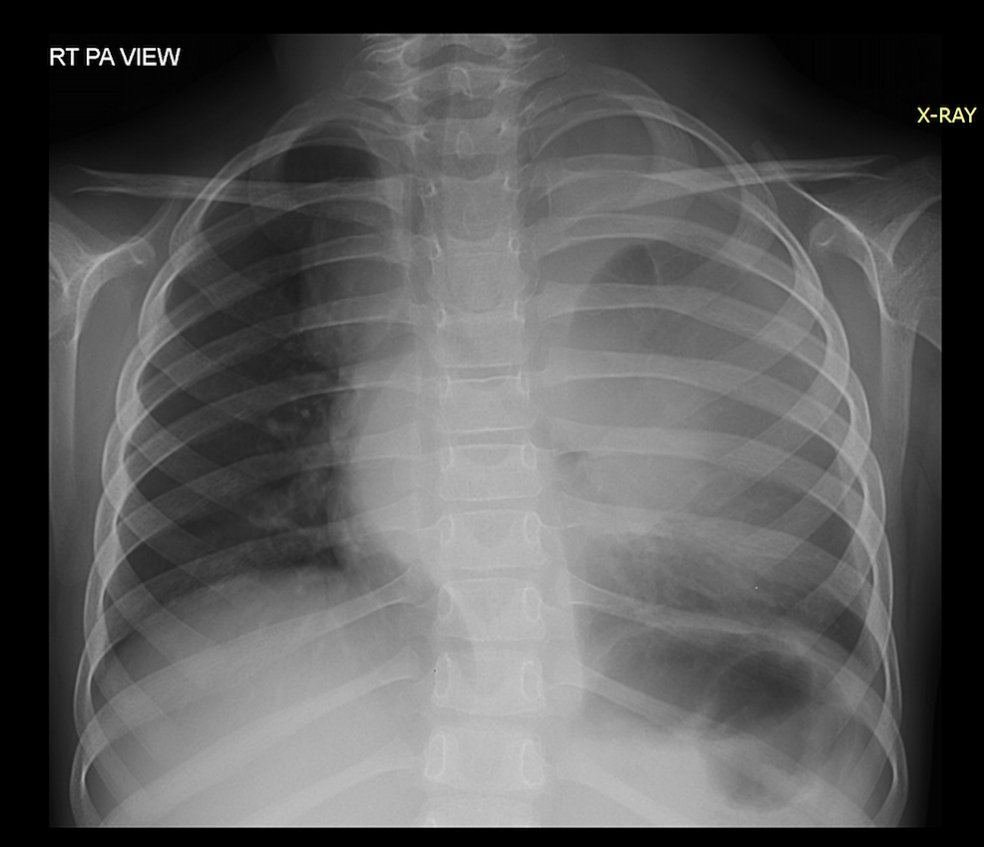
Pre-chemotherapy mediastinal mass on chest x-ray. Middle and left-sided haziness on chest x-ray representing a mediastinal mass.

Physical examination was significant for a 5 cm swelling in her left neck and palpable left-sided non-tender, matted, and rubbery supraclavicular lymphadenopathy with freely mobile and smooth overlying skin. There was reduced air entry on the left side with no wheezing or added sounds on chest examination. The rest of the physical examination was unremarkable. The baseline complete blood count test showed mild neutrophilia and thrombocytosis as the only abnormalities. Her chest x-ray revealed a large mediastinal mass (Figure 1). Other baseline investigations, including urea, creatinine, urine analysis, and hepatic function panel, were within normal limits. The patient was hospitalized for an excisional biopsy of the left supraclavicular lymph node, and the histopathology report confirmed a classical Hodgkin lymphoma of mixed cellularity type.

**Figure 2:**
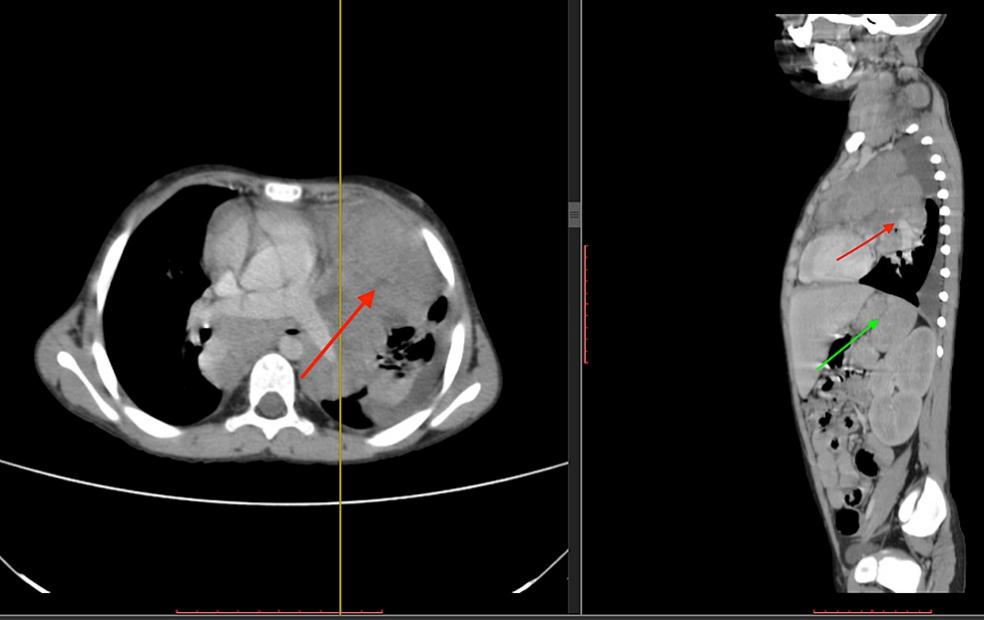
CT Neck, Chest, Abdomen, and Pelvis with Contrast A bulky mediastinal mass causing left lung upper lobe collapse (red arrow) and minor left-sided pleural effusion along with the involvement of spleen (green arrow).

To further investigate for spread and appropriate staging, she was scheduled for a bone marrow biopsy, CT, and positron emission tomography (PET) scan. The biopsy report did not reveal any evidence of bone marrow involvement. However, staging CT and PET scan reports concluded a bulky mediastinal nodal disease which caused left lung upper lobe collapse and minor left-sided pleural effusion, along with the involvement of spleen and splenic hilar nodes (Figure 2). Although imaging did not clearly exhibit hypodense renal lesions, both kidneys were also suspected to be involved in the disease. Based on these results, a diagnosis of stage 4 Hodgkin's lymphoma was made. 

**Figure 3:**
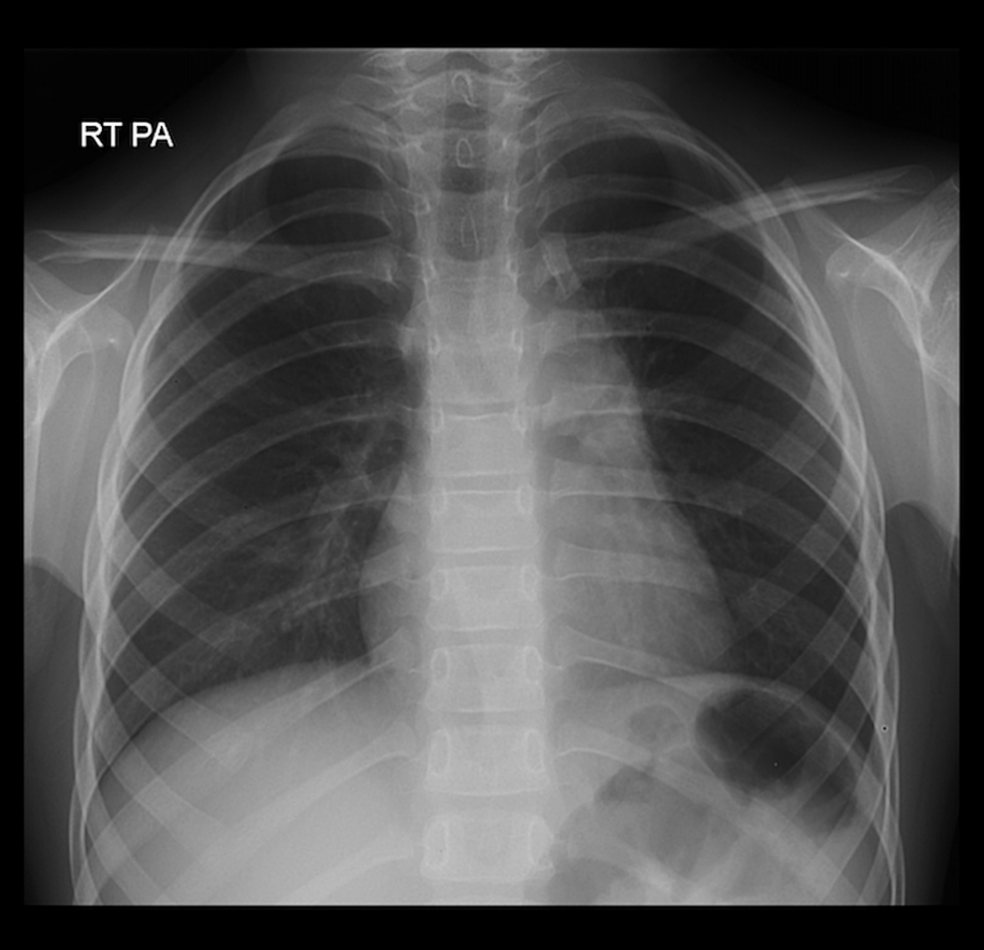
Post-chemotherapy chest x-ray Significant reduction in the size of the mediastinal mass demonstrated by well-aerated (black) lungs on the chest x-ray

The patient was admitted to the hospital for the treatment of malignant lymphoma. On the second day of hospitalization, before initiation of chemotherapy, she was vitally and clinically stable with the exception of mild dehydration (dry oral mucous membranes with the capillary refill of 2-3 seconds, and normal urinary output). Her laboratory findings were consistent with metabolic acidosis (HCO3 16mEq/L, Normal Range 22-28 mEq/L), elevated LDH (496U/L, Normal range 120-200), hyperuricemia (10 mg/dl, Normal range 2-6mg/dl), hyperkalemia (5.74 mmol/L, Normal range 3.3-5.1), hypocalcemia (8.73 mg/dl, Normal range 8.8-10.8), and hyperphosphatemia (7 mg/dl, Normal range 2.5-6.0). The diagnosis of spontaneous tumor lysis syndrome was suspected (extremely rare in patients with Hodgkin's lymphoma), and she immediately received intravenous fluids, allopurinol, urine alkalization, and forced diuresis therapy. The patient responded well to the treatment and recovered without developing further complications. Finally, under oncology care, the patient received multiple cycles of chemotherapy (ABVE-PC Regimen) followed by radiation therapy. The mediastinal mass showed a significant reduction in size after receiving chemotherapy (Figure 3). The blood counts returned to normal levels within three months.

## Discussion

Spontaneous tumor lysis syndrome is a rare occurrence in hematological malignancies. According to previous studies, the risk of tumor lysis syndrome after chemotherapy administration in hematological cancers is 4-42%, and the incidence of STLS in patients diagnosed with hematological malignancies is only 1.08% [2]. Another study revealed that among 33 patients diagnosed with non-Hodgkin’s lymphoma, only three patients developed STLS [3]. Patients with bulky tumors, high pretreatment uric acid levels, preexisting kidney diseases, exposure to nephrotoxins, oliguria, acidic urine, and dehydration are at increased risk of developing STLS [2]. In our case, patient factors such as the preexisting kidney disease, bulky tumor, enlarged mediastinal mass, and dehydration put her at high risk of developing STLS [4].

Cairo and Bishop Criteria includes the laboratory findings of uric acid level>8 mg/dL, potassium>6 mg/dL, phosphorus >4.5 mg/dL, calcium<7 mg/dL, and clinical symptoms of impaired renal function (creatinine>1.5 times of upper limit of normal), cardiac arrhythmia/sudden death, new-onset seizures. To diagnose Laboratory TLS, abnormality in two or more of the above-mentioned labs within three days prior or seven days after starting chemotherapy should be present. For clinical TLS, there should be laboratory TLS plus one of the clinical symptoms [5]. However, this criteria is insufficient to diagnose spontaneous TLS, and according to one study, even underestimates the incidence of STLS [6]. A retrospective study done in 2004 studied 926 patients with acute renal failure and based the diagnosis of STLS on the presence of uric acid nephropathy, elevated LDH, and biopsy-proven malignancy instead of completely focusing on metabolic and clinical criteria [6]. In the presented case, metabolic acidosis, increased LDH, hyperuricemia, hyperkalemia, hypocalcemia, and hyperphosphatemia were present, suggesting the presence of STLS. 

Mediastinum involvement is commonly seen in patients with Hodgkin’s disease; the obstructive airway symptoms are rare. The CRC Wessex Regional Medical Oncology Unit did a case series study between 1978-1989 reported that out of 254 newly diagnosed cases of Hodgkin’s disease during that period, only 2.4% of patients presented with early-onset obstructive symptoms due to the mediastinal mass [7]. Despite the extensive involvement of mediastinum with a large bulky mass seen on the CT scan, our patient did not manifest any obstructive symptoms except for the chronic cough.

## Conclusions

The etiology of spontaneous tumor lysis syndrome is purely uncertain at this point. STLS, in the case of Hodgkin’s disease, is an infrequent phenomenon. A different hypothesis has been proposed for the etiology of STLS, including increased cytokines, glucocorticoids, and hyperthermia, all of which can lead to increased tumor cell death. More research needs to be done in this field to understand the etiology and pathophysiology of STLS better. In fact, STLS is rare, but if developed, it has a high risk for worse clinical outcomes due to the physician’s perceived underestimation of its occurrence and failure to initiate timely treatment. Oftentimes, the vague and nonalarming nature of complaints makes this easily treated condition difficult to diagnose, which triggers a series of fatal consequences. The objective of writing this case report is to prompt the attention of general physicians towards the rare yet important occurrence of STLS with Hodgkin lymphoma cases.
